# Symptom‐Domain Correlates of Self‐Reported Anxious Distress in Outpatients With Major Depressive Disorder

**DOI:** 10.1002/npr2.70165

**Published:** 2026-09-01

**Authors:** Shinpei Oga, Norio Sugawara, Yasushi Kawamata, Tomohiro Asahi, Yujin Ono, Sakurako Suzuki, Rina Hiramatsu, Norio Yasui‐Furukori

**Affiliations:** ^1^ Department of Psychiatry Dokkyo Medical University Mibu Tochigi Japan

**Keywords:** anxious distress, depressive symptoms, major depressive disorder, QIDS‐J, symptom domains

## Abstract

**Aim:**

Anxious distress is common in major depressive disorder (MDD), but real‐world data are limited on its burden and relationship to depressive symptom domains. This study examined the prevalence and symptom‐domain correlates of self‐reported anxious distress in MDD outpatients.

**Methods:**

This cross‐sectional study used routinely collected questionnaire and clinical data from Dokkyo Medical University Hospital. Depressive severity was assessed using the Japanese version of the Quick Inventory of Depressive Symptomatology (QIDS‐J). Anxious distress was assessed using five self‐rated items aligned with the Clinically Useful Depression Outcome Scale‐Anxious Distress (CUDOS‐A) and DSM‐5 framework. Of 309 respondents, the primary analytic cohort comprised 206 participants with QIDS‐J total scores of 6 or higher and complete anxious‐distress item data.

**Results:**

A lower anxious‐distress threshold (at least two items scored > = 2) was met by 153 participants (74.3%), and a stringent threshold (at least two items scored > = 3) by 85 (41.3%). QIDS‐J total score correlated with anxious‐distress burden (*r* = 0.614 for > = 2 count, *r* = 0.669 for > = 3 count, and *r* = 0.697 for summed score; all *p* < 0.001). In forced‐entry Poisson models, sad mood, negative self‐view, and psychomotor symptoms were associated with anxious‐distress burden. Post hoc decomposition suggested that this psychomotor association was driven mainly by psychomotor agitation rather than retardation.

**Conclusion:**

Self‐reported anxious distress was common among MDD outpatients and was closely associated with depressive severity and specific symptom domains, supporting further evaluation of anxious distress as a clinically relevant psychopathological dimension in MDD.

## Abstract 2

In outpatients with major depressive disorder, self‐reported anxious distress was common and increased with depressive symptom severity. Sad mood, negative self‐view, and psychomotor symptoms showed the most consistent associations, supporting anxious distress as a clinically relevant symptom dimension.

## Graphical abstract

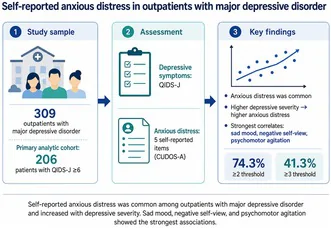

**Abbreviations**
CI: confidence interval
CUDOS: Clinically Useful Depression Outcome Scale
CUDOS‐A: Clinically Useful Depression Outcome Scale‐Anxious Distress
HC3: heteroskedasticity‐consistent type 3
IRR: incidence rate ratio
MDD: major depressive disorder
QIDS‐J: Japanese version of the Quick Inventory of Depressive Symptomatology
SD: standard deviation
VIF: variance inflation factor

## 1 Introduction

Anxious distress is a clinically important dimension of major depressive disorder (MDD) and is recognized in DSM‐5 as a specifier for depressive disorders [1]. Recent Japanese clinical practice guidance has also emphasized consideration of anxious distress when initiating treatment for depression [2]. Prior studies indicate that anxious distress is common among patients with MDD and identifies a subgroup with greater depressive severity, lower remission rates, greater treatment complexity, and elevated suicide‐related risk [3, 4, 5, 6, 7, 8, 9, 10, 11, 12]. Because anxious distress captures tension, restlessness, worry‐related concentration difficulty, fear that something awful may happen, and fear of losing control, it may represent a clinically meaningful symptom dimension that is not fully captured by depressive severity alone.

Anxious distress should not be equated with a comorbid anxiety disorder. Although anxiety disorders frequently co‐occur with MDD [13, 14], the anxious‐distress specifier describes anxiety‐related symptoms occurring within the depressive disorder itself. Previous work suggests that this dimension may remain relevant even without a primary anxiety disorder and during continuation or maintenance treatment [15, 16]. This distinction is clinically important because routine MDD care includes both acutely symptomatic patients and patients in remission receiving maintenance treatment.

Measurement‐based assessment has been advocated to improve systematic evaluation of depressive symptoms and outcomes [17, 18, 19, 20]. Brief self‐administered questionnaires may help patients report symptoms that may not be volunteered spontaneously [21]. The Clinically Useful Depression Outcome Scale (CUDOS) and related anxious‐distress assessments were developed to support routine evaluation, and Japanese psychometric data are available for a CUDOS version supplemented with DSM‐5 anxious‐distress items [22, 23, 24, 25]. However, real‐world data remain limited on the prevalence and depressive symptom‐domain correlates of self‐reported anxious distress in pragmatic MDD outpatient workflows. This study examined the prevalence and clinical correlates of anxious distress in outpatients with MDD, focusing on associations with current depressive symptom severity as assessed by the Japanese version of the Quick Inventory of Depressive Symptomatology (QIDS‐J) and individual QIDS‐J symptom domains.

## 2 Methods

### 2.1 Study Design and Participants

This cross‐sectional observational study used routinely collected questionnaire and clinical data from psychiatric outpatient care at a single center in Japan. The questionnaire was administered to patients with a clinical diagnosis of MDD, including those currently experiencing a depressive episode and those in remission receiving maintenance treatment. Data collected from the questionnaire and clinical record included age, sex, clinical diagnosis, QIDS‐J items, and five anxious‐distress items aligned with the DSM‐5/CUDOS‐A framework. The questionnaire was part of routine clinical care rather than an anonymous research survey. The present study was conducted as a secondary‐use analysis of routinely collected clinical data.

Of the 309 respondents, 19 had missing QIDS‐J data and 16 had incomplete anxious‐distress item data. A total of 287 participants had complete data for both measures. Among the 209 participants with QIDS‐J total scores of 6 or higher, three had incomplete anxious‐distress item data, yielding a primary analytic sample of 206 participants. The QIDS‐J threshold of 6 was selected because QIDS‐J total scores of 0–5 are generally interpreted as none/minimal depressive symptoms or remission, whereas scores of 6 or higher indicate at least mild current depressive symptom burden [26, 27, 28]. This restriction therefore focused the primary analyses on patients with current clinically relevant depressive symptoms, while descriptive results for the broader screened cohort were retained. Clinical diagnoses were made in routine practice and were not established using structured diagnostic interviews for research purposes.

This study was approved by the Ethics Committee of Dokkyo Medical University Hospital (R‐106‐5 J). All methods were performed in accordance with relevant guidelines and regulations, including the Declaration of Helsinki. Because this research involved the secondary use of routinely collected clinical data, an opt‐out approach was used to provide patients with an opportunity to refuse participation. The requirement for individual written informed consent was waived by the Ethics Committee of Dokkyo Medical University Hospital.

### 2.2 Measures

Depressive symptom severity was assessed using the QIDS‐J, a self‐report measure scored using the standard QIDS algorithm [26, 27, 28]. The QIDS‐J assesses nine depressive symptom domains: sleep, sad mood, appetite and weight, concentration and decision making, self‐view, suicidal ideation, general interest, energy and fatigue, and psychomotor symptoms. The QIDS‐J psychomotor domain is scored using two raw items, psychomotor retardation and psychomotor agitation, with the higher of the two item scores used as the domain score.

Anxious distress was assessed using five self‐rated items embedded in the local depression questionnaire and aligned with the DSM‐5/CUDOS‐A anxious‐distress framework [1, 23, 24, 25]. Each item was scored on a 0–4 scale. The present analysis used only these five anxious‐distress items; the full CUDOS depression scale was not analyzed as an outcome. Two prespecified thresholds were used for descriptive and analytic purposes. The lower threshold was defined as at least two items scored > = 2, reflecting the DSM‐5 symptom‐count requirement of at least two anxious‐distress symptoms and item‐level severity ratings in the CUDOS‐A framework. The stringent threshold was defined as at least two items scored > = 3 and was used as a prespecified exploratory threshold for more intense anxious‐distress burden. The summed anxious‐distress score ranged from 0 to 20. These thresholds were used to characterize symptom burden and were not intended to establish diagnostic accuracy.

### 2.3 Statistical Analysis

Continuous variables are presented as mean and standard deviation (SD), and categorical variables as number and percentage. Complete‐case analysis was used because the proportion of missing data was low. The primary association analysis examined the Pearson correlation between QIDS‐J total score and the number of anxious‐distress items scored > = 3 in the primary analytic cohort. Secondary correlation analyses used the number of items scored > = 2 and the summed anxious‐distress score. Supplementary domain‐level analyses evaluated correlations between each QIDS‐J symptom domain and anxious‐distress burden; Benjamini‐Hochberg false‐discovery‐rate correction was applied.

Group comparisons between threshold‐positive and threshold‐negative participants were performed using Welch *t*‐tests for continuous variables and chi‐square tests for sex. Forced‐entry Poisson regression models with HC3 robust standard errors were fitted using all nine QIDS‐J domains, age per 10 years, and sex as explanatory variables. Two count outcomes were modeled separately: the number of anxious‐distress items scored > = 2 and the number scored > = 3. Model fit and possible overdispersion were assessed using Pearson chi‐square divided by residual degrees of freedom and deviance divided by residual degrees of freedom. Multicollinearity was assessed using variance inflation factors (VIFs). Because the QIDS‐J psychomotor domain combines psychomotor retardation and psychomotor agitation, post hoc supplementary analyses decomposed this domain into the two raw items and repeated correlations and adjusted Poisson models. Two‐sided *p* values < 0.05 were considered statistically significant.

## 3 Results

### 3.1 Sample Characteristics and Missing Data

**FIGURE 1:**
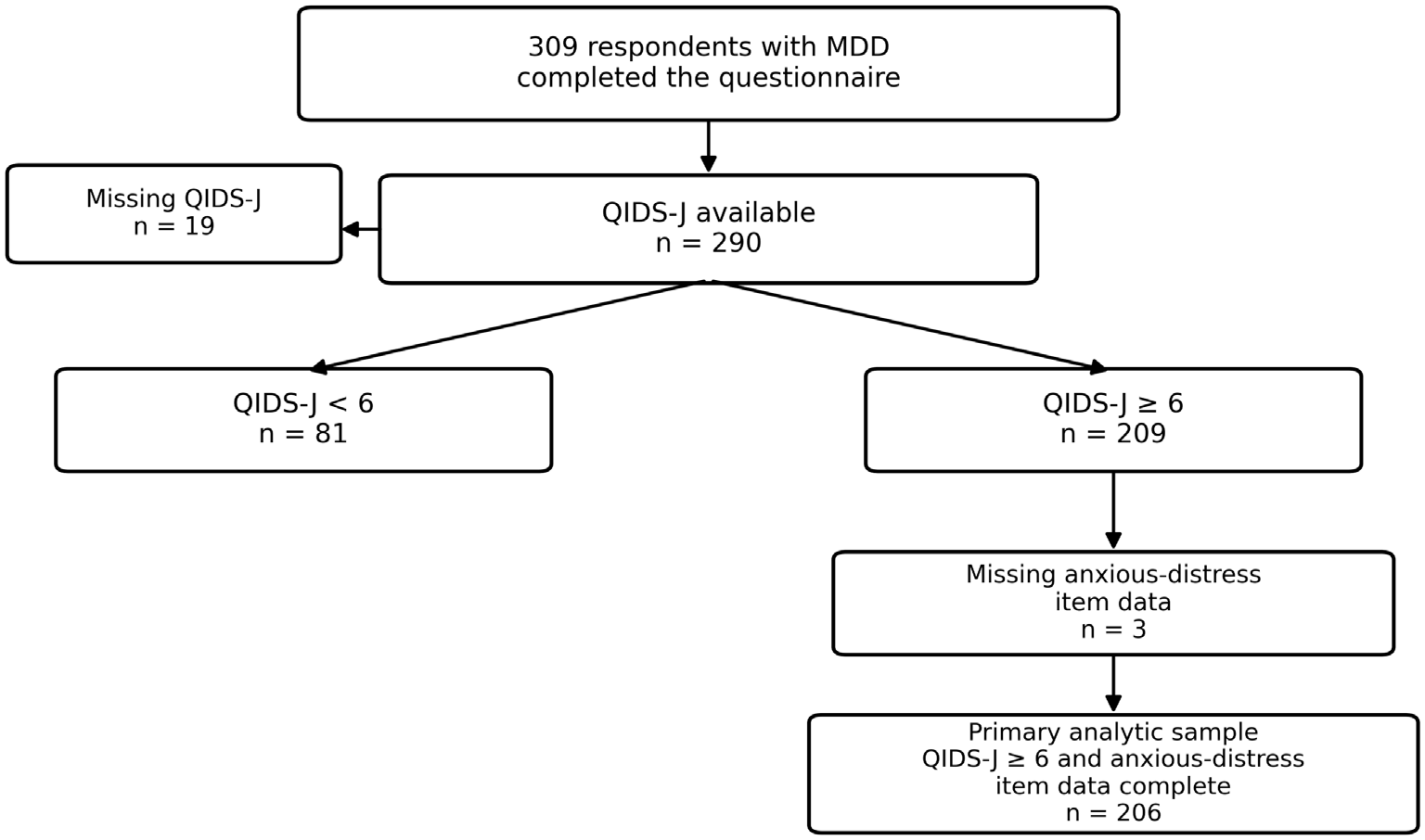
Study flow diagram. Of the 309 respondents, 19 had missing QIDS‐J data and 16 had incomplete anxious‐distress item data. Among the 209 participants with QIDS‐J total scores of 6 or higher, three had incomplete anxious‐distress item data, yielding a primary analytic sample of 206 participants.

Of the 309 respondents, 19 had missing QIDS‐J data and 16 had incomplete anxious‐distress item data. A total of 287 participants had complete data for both measures. Among the 209 participants with QIDS‐J total scores of 6 or higher, three had incomplete anxious‐distress item data, yielding a primary analytic sample of 206 participants (Figure 1).

In the full screened cohort, the prevalence of the lower anxious‐distress threshold was 163 of 293 (55.6%), and the prevalence of the stringent anxious‐distress threshold was 86 of 293 (29.4%). In the primary analytic cohort, mean age was 45.1 (SD 16.0) years, 130 of 206 participants (63.1%) were women, and mean QIDS‐J total score was 13.6 (SD 5.4). The lower anxious‐distress threshold was met by 153 of 206 participants (74.3%), and the stringent threshold was met by 85 of 206 participants (41.3%) (Table 1). Across QIDS‐J severity strata, anxious‐distress burden increased monotonically with depressive severity (Table S4).

**TABLE 1:** Characteristics of the study cohorts and missing data.

Characteristic	Full screened cohort (*n* = 309)	QIDS‐J + anxious‐distress item complete cohort (*n* = 287)	Primary analytic cohort (QIDS‐J > =6; *n* = 206)
*N*	309	287	206
Age, years	47.4 ± 17.3	46.6 ± 16.8	45.1 ± 16.0
Female sex	187/309 (60.5%)	175/287 (61.0%)	130/206 (63.1%)
QIDS‐J total score	10.5 ± 6.8	10.5 ± 6.8	13.6 ± 5.4
Anxious‐distress summed score	7.3 ± 6.0	7.3 ± 6.0	9.5 ± 5.6
Lower anxious‐distress threshold (> = 2 items scored > = 2)	163/293 (55.6%)	161/287 (56.1%)	153/206 (74.3%)
Stringent anxious‐distress threshold (> = 2 items scored > = 3)	86/293 (29.4%)	86/287 (30.0%)	85/206 (41.3%)

*Note:* Data are presented as mean ± SD or number/total (%). The complete‐case cohort includes participants with both QIDS‐J and anxious‐distress item data available. The analytic cohort consists of participants with QIDS‐J total scores of 6 or higher and complete anxious‐distress item data. Missing data were as follows: QIDS‐J, *n* = 19; anxious‐distress item data, *n* = 16.

Abbreviations: QIDS‐J, Japanese version of the Quick Inventory of Depressive Symptomatology; SD, standard deviation.

### 3.2 Correlations and Symptom‐Domain Analyses

**FIGURE 2:**
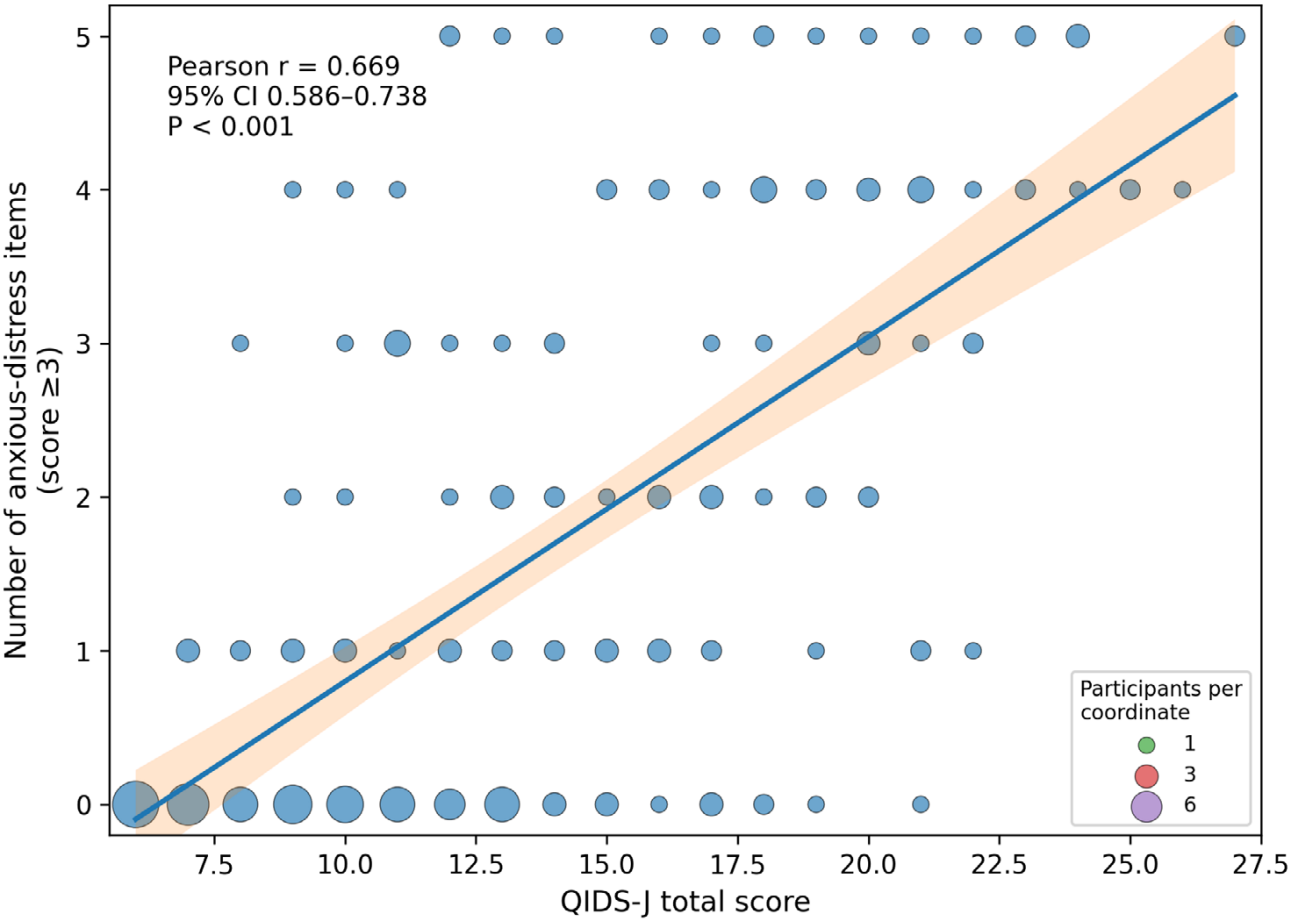
Association between depressive severity and anxious‐distress burden. Bubble scatter plot of QIDS‐J total score versus the number of anxious‐distress items scored > = 3 in the primary analytic cohort (*n* = 206). Each point represents a unique coordinate, and point size indicates the number of participants with the same QIDS‐J/anxious‐distress count combination. The solid line shows the linear fit and the shaded area the 95% confidence interval.

QIDS‐J total score correlated significantly with the number of anxious‐distress items scored > = 2 (*r* = 0.614, 95% CI 0.521–0.693, *p* < 0.001), the number scored > = 3 (*r* = 0.669, 95% CI 0.586–0.738, *p* < 0.001), and the summed anxious‐distress score (*r* = 0.697, 95% CI 0.620–0.761, *p* < 0.001) (Figure 2).

In supplementary domain‐level analyses, all nine QIDS‐J symptom domains were positively correlated with anxious‐distress burden, and all associations remained significant after false‐discovery‐rate correction (Tables S1–S3). For the primary anxious‐distress metric, defined as the number of items scored > = 3, the strongest correlations were observed for sad mood (*r* = 0.591), self‐view (*r* = 0.517), suicidal ideation (*r* = 0.508), psychomotor symptoms (*r* = 0.504), and concentration and decision making (*r* = 0.468).

### 3.3 Group Comparisons and Multivariable Models

Compared with participants who did not meet the lower anxious‐distress threshold, those who did were younger, more often female, and had higher total QIDS‐J scores. Participants meeting the more stringent threshold were younger and had substantially higher depressive severity, whereas sex differences were not significant in unadjusted comparisons. Scores for all nine QIDS‐J domains were higher in threshold‐positive groups for both thresholds (Table 2).

**TABLE 2:** Comparison of clinical characteristics by anxious‐distress thresholds.

Variable	Threshold > = 2 positive (*n* = 153)	Threshold > = 2 negative (*n* = 53)	*p*	Threshold > = 3 positive (*n* = 85)	Threshold > = 3 negative (*n* = 121)	*p*
Age	43.4 ± 15.1	49.8 ± 17.7	0.021	41.3 ± 13.1	47.7 ± 17.3	0.003
Female sex	103/153 (67.3%)	27/53 (50.9%)	0.049	58/85 (68.2%)	72/121 (59.5%)	0.258
QIDS‐J total	15.1 ± 5.1	9.4 ± 3.3	< 0.001	17.5 ± 4.6	10.9 ± 4.0	< 0.001
Sleep	2.4 ± 0.8	2.0 ± 1.0	0.004	2.6 ± 0.7	2.1 ± 0.9	< 0.001
Sad mood	1.6 ± 0.9	0.5 ± 0.6	< 0.001	1.9 ± 0.9	0.9 ± 0.7	< 0.001
Appetite/weight	1.8 ± 1.1	1.5 ± 1.1	0.036	2.0 ± 1.0	1.5 ± 1.1	0.001
Concentration/decision making	1.4 ± 0.9	0.8 ± 0.7	< 0.001	1.7 ± 0.9	0.9 ± 0.7	< 0.001
Self‐view	1.9 ± 0.9	1.2 ± 0.9	< 0.001	2.3 ± 0.8	1.4 ± 0.9	< 0.001
Suicidal ideation	1.3 ± 1.0	0.5 ± 0.7	< 0.001	1.6 ± 1.0	0.7 ± 0.8	< 0.001
General interest	1.6 ± 1.1	1.1 ± 1.0	0.001	1.9 ± 1.0	1.2 ± 1.0	< 0.001
Energy/fatigue	1.6 ± 0.8	1.1 ± 0.7	< 0.001	1.7 ± 0.8	1.3 ± 0.8	< 0.001
Psychomotor	1.4 ± 0.9	0.7 ± 0.7	< 0.001	1.7 ± 0.9	0.9 ± 0.7	< 0.001

*Note:* Values are mean ± SD or number/total (%). *p* values were calculated using Welch *t*‐tests for continuous variables and chi‐square tests for sex.

Abbreviations: QIDS‐J, Japanese version of the Quick Inventory of Depressive Symptomatology; SD, standard deviation.

In forced‐entry Poisson regression models, sad mood, negative self‐view, and psychomotor symptoms were independently associated with both count outcomes. For the number of anxious‐distress items scored > = 2, significant associations were observed for sad mood (IRR 1.25), self‐view (IRR 1.20), psychomotor symptoms (IRR 1.12), and female sex (IRR 1.18). For the number of items scored > = 3, sleep disturbance (IRR 1.33), sad mood (IRR 1.48), self‐view (IRR 1.52), psychomotor symptoms (IRR 1.28), female sex (IRR 1.30), and an inverse association for energy and fatigue (IRR 0.77) were observed (Table 3). Age was not independently associated after simultaneous adjustment.

**TABLE 3:** Forced‐entry Poisson regression models for anxious‐distress burden.

Predictor	Count > = 2 IRR (95% CI)	*p*	Count > = 3 IRR (95% CI)	*p*
Sleep	1.07 (0.96–1.18)	0.217	1.33 (1.10–1.62)	0.004
Sad mood	1.25 (1.15–1.36)	< 0.001	1.48 (1.27–1.73)	< 0.001
Appetite/weight	1.07 (0.99–1.15)	0.095	1.06 (0.93–1.21)	0.370
Concentration/decision making	1.01 (0.92–1.11)	0.854	1.11 (0.94–1.31)	0.233
Self‐view	1.20 (1.09–1.32)	< 0.001	1.52 (1.26–1.83)	< 0.001
Suicidal ideation	0.99 (0.91–1.08)	0.871	1.01 (0.85–1.21)	0.901
General interest	1.02 (0.94–1.11)	0.631	1.04 (0.90–1.19)	0.610
Energy/fatigue	0.97 (0.88–1.07)	0.515	0.77 (0.66–0.90)	0.001
Psychomotor	1.12 (1.03–1.21)	0.006	1.28 (1.10–1.48)	0.001
Age, per 10 years	1.00 (0.95–1.06)	0.892	0.93 (0.86–1.01)	0.073
Female sex	1.18 (1.00–1.38)	0.043	1.30 (1.02–1.66)	0.037

*Note:* All nine QIDS‐J symptom domains, age, and sex were entered simultaneously into the models. Estimates are incidence rate ratios (IRR) with 95% confidence intervals based on HC3 robust standard errors. VIFs ranged from 1.04 to 1.82. Pearson chi‐square/residual df and deviance/residual df were 0.96 and 1.25 for the count > = 2 model and 1.17 and 1.23 for the count > = 3 model, respectively.

Abbreviations: CI, confidence interval; HC3, heteroskedasticity‐consistent type 3; IRR, incidence rate ratio; QIDS‐J, Japanese version of the Quick Inventory of Depressive Symptomatology; VIF, variance inflation factor.

Model diagnostics supported the use of Poisson models (Table S6). Pearson chi‐square divided by residual degrees of freedom and deviance divided by residual degrees of freedom were 0.96 and 1.25, respectively, for the count > = 2 model, and 1.17 and 1.23 for the count > = 3 model. VIFs ranged from 1.04 to 1.82, indicating no evidence of problematic multicollinearity among the predictors.

In post hoc analyses decomposing the QIDS‐J psychomotor domain, both psychomotor retardation and psychomotor agitation were positively correlated with anxious‐distress burden. However, when both items were entered simultaneously with the other QIDS‐J domains, age and sex, psychomotor agitation remained independently associated with both anxious‐distress count outcomes, whereas psychomotor retardation did not (Table S5).

## 4 Discussion

This study examined self‐reported anxious distress in routine outpatient care for patients with MDD. The workflow included not only patients in an active depressive episode but also patients in remission receiving maintenance treatment. More than half of respondents with available anxious‐distress item data met the lower anxious‐distress threshold, and nearly three quarters of the symptomatic analytic cohort did so. These findings are consistent with prior work showing that anxious distress is common in MDD and is associated with greater clinical burden [3, 4, 5, 6, 7, 8, 9, 10, 11, 12, 13, 14, 15, 16, 29, 30].

The present findings also extend Japanese real‐world studies from our group that examined depressive symptom trajectories, suicidal ideation, working ability, and clinical characteristics of patients with MDD [31, 32, 33, 34]. Compared with these previous reports, the present study focuses specifically on self‐reported anxious distress and its association with individual QIDS‐J symptom domains, adding a symptom‐domain perspective to routine outpatient assessment of MDD.

Across both count outcomes, sad mood, negative self‐view, and the composite psychomotor domain were the most consistent independent correlates of anxious‐distress burden. This pattern suggests that anxious distress in MDD is not merely a nonspecific reflection of total depressive severity. Sad mood may represent the affective core of the depressive state, whereas negative self‐view may capture self‐critical or hopeless appraisals that amplify worry, perceived loss of control, and anticipatory threat.

The interpretation of psychomotor symptoms requires particular caution. The QIDS‐J psychomotor domain is a composite domain based on psychomotor retardation and psychomotor agitation, and these two phenomena may reflect different psychopathological processes. This distinction is especially relevant because motor agitation is included in the DSM‐5 anxious distress specifier as an indicator of severe anxious distress, whereas psychomotor retardation may not have the same clinical implication. In our post hoc decomposition, psychomotor agitation remained independently associated with anxious‐distress item count after adjustment for the other depressive symptom domains, age, and sex, whereas psychomotor retardation did not. Thus, the association observed for the composite psychomotor domain should be interpreted primarily as reflecting agitation/restlessness‐related features rather than psychomotor disturbance as a whole.

In supplementary correlations, suicidal ideation was strongly associated with anxious‐distress burden, although it was not retained as an independent correlate in the forced‐entry multivariable models. This likely reflects shared variance with sad mood and negative self‐view. Clinically, patients with prominent anxious distress should still receive careful assessment of suicidal ideation, agitation, impulsive deterioration, and perceived loss of control. For the more stringent anxious‐distress count, sleep disturbance and female sex were additionally associated. The inverse association for energy and fatigue should be interpreted cautiously because it may reflect a suppression effect in a fully adjusted model rather than a direct protective relationship.

These findings suggest that anxious distress represents a clinically meaningful psychopathological dimension within MDD. Nevertheless, the present study was cross‐sectional and was designed to describe prevalence and clinical correlates, not diagnostic accuracy, prediction, causality, or formal psychometric validation. Additional limitations include the single‐center design, routine clinical rather than structured diagnostic assessment, complete‐case analysis, self‐rated anxious‐distress items rather than clinician‐administered interviews, and the absence of detailed medication, episode duration, comorbidity, functioning, relapse, hospitalization, or longitudinal suicide‐related outcome data. In addition, although post hoc analyses separated psychomotor agitation from retardation, these analyses were based on individual QIDS‐J items and should be considered exploratory. Future studies should integrate anxious‐distress assessment into longitudinal measurement‐based care frameworks to determine whether anxious‐distress burden contributes to changes in depressive symptoms, suicidal ideation, functioning, and treatment outcomes over time.

## 5 Conclusion

Self‐reported anxious distress was highly prevalent among outpatients with MDD and was closely associated with depressive severity and specific symptom domains, especially sad mood, negative self‐view, and psychomotor agitation‐related features. These findings support further investigation of anxious distress as a clinically relevant psychopathological dimension within MDD.

## Author Contributions

All authors made a significant contribution to the work reported, including study conception, study design, execution, acquisition of data, analysis, or interpretation. N.Y.‐F. conceived and designed the study. S.O. drafted the manuscript. N.S. performed the statistical analyses. All authors critically reviewed or revised the manuscript, agreed on the journal to which the article will be submitted, approved the final version, and agree to take responsibility and be accountable for the contents of the article.

## Funding

The authors have nothing to report.

## Ethics Statement

This study was approved by the Ethics Committee of Dokkyo Medical University Hospital (R‐106‐5 J). All methods were performed in accordance with relevant guidelines and regulations, including the Declaration of Helsinki. Because this research involved the secondary use of routinely collected clinical data, an opt‐out approach was used to provide patients with an opportunity to refuse participation. The requirement for individual written informed consent was waived by the Ethics Committee of Dokkyo Medical University Hospital.

## Conflicts of Interest

N.Y.‐F. reports having received honoraria for lectures from Daiichi Sankyo, EA Pharma, Eisai, Eli Lilly and Company, Hisamitsu Pharmaceutical, Janssen Pharmaceuticals, Kowa, Lundbeck, Meiji Seika Pharma, Mitsubishi Tanabe Pharma, Mochida Pharmaceutical, MSD, Ono Pharmaceutical, Otsuka Pharmaceutical, Shionogi, Sumitomo Pharma, Takeda Pharmaceutical, Tsumura, Viatris, and Yoshitomi Yakuhin; has served in advisory or consultancy roles for EA Pharma, Bristol Myers Squibb, Boehringer Ingelheim, Janssen Pharmaceuticals, Mitsubishi Tanabe Pharma, Mochida Pharmaceutical, Sumitomo Pharma, and Otsuka Pharmaceutical; and has participated as a sub‐investigator in industry sponsored clinical trials conducted by EA Pharma and Bristol Myers Squibb. The remaining authors declare no conflicts of interest.

## Supporting information


**Table S1:** Correlations between QIDS‐J symptom domains and anxious‐distress burden (> = 3 threshold).
**Table S2:** Correlations between QIDS‐J symptom domains and anxious‐distress burden (> = 2 threshold).
**Table S3:** Correlations between QIDS‐J symptom domains and total anxious‐distress score.
**Table S4:** Distribution of anxious‐distress burden across QIDS‐J severity categories.
**Table S5:** Post hoc decomposition of the QIDS‐J psychomotor domain.
**Table S6:** Model diagnostics for forced‐entry Poisson regression models.

## Data Availability

All summary data supporting the findings of this study are included within the article and its Tables S1–S6. The individual participant‐level datasets used and/or analyzed during the current study are not publicly available because they were derived from routinely collected clinical records and questionnaire data from psychiatric outpatients at a single institution. Even after removal of direct identifiers, the dataset contains potentially identifying combinations of demographic and clinical information and symptom‐item responses. In addition, the present study was approved as a secondary‐use analysis of routine clinical data using an opt‐out approach; therefore, public deposition of participant‐level raw data was outside the scope of the approved opt‐out procedure. Qualified researchers may request access to a de‐identified, limited dataset from the corresponding author. Such requests will be considered on a case‐by‐case basis and will require appropriate ethical approval by the Ethics Committee of Dokkyo Medical University Hospital and, where applicable, a data‐use agreement.

## References

[1] American Psychiatric Association , ed., Diagnostic and Statistical Manual of Mental Disorders, 5th ed. (American Psychiatric Association, 2013).

[2] Japanese Society of Mood Disorders , Utsubyo Shinryo Guideline 2025 [Depression Clinical Practice Guideline 2025] (Japanese Society of Mood Disorders, 2025), https://www.secretariat.ne.jp/jsmd/iinkai/katsudou/data/guideline2025.pdf.

[3] D. S. Hasin , A. L. Sarvet , J. L. Meyers , et al., “Epidemiology of Adult DSM‐5 Major Depressive Disorder and Its Specifiers in the United States,” JAMA Psychiatry 75, no. 4 (2018): 336–346, 10.1001/jamapsychiatry.2017.4602. 29450462 PMC5875313

[4] R. S. McIntyre , H. O. Woldeyohannes , J. K. Soczynska , et al., “The Prevalence and Clinical Characteristics Associated With Diagnostic and Statistical Manual Version‐5‐Defined Anxious Distress Specifier in Adults With Major Depressive Disorder: Results From the International Mood Disorders Collaborative Project,” Therapeutic Advances in Chronic Disease 7, no. 3 (2016): 153–159, 10.1177/2040622315627805. 27347362 PMC4907069

[5] A. J. Rosellini , M. L. Bourgeois , J. Correa , E. S. Tung , S. Goncharenko , and T. A. Brown , “Anxious Distress in Depressed Outpatients: Prevalence, Comorbidity, and Incremental Validity,” Journal of Psychiatric Research 103 (2018): 54–60, 10.1016/j.jpsychires.2018.05.006. 29778071 PMC8903047

[6] M. Zimmerman , J. Martin , P. McGonigal , et al., “Validity of the DSM‐5 Anxious Distress Specifier for Major Depressive Disorder,” Depression and Anxiety 36, no. 1 (2019): 31–38, 10.1002/da.22837. 30311733

[7] R. Gaspersz , F. Lamers , J. M. Kent , et al., “Anxious Distress Predicts Subsequent Treatment Outcome and Side Effects in Depressed Patients Starting Antidepressant Treatment,” Journal of Psychiatric Research 84 (2017): 41–48, 10.1016/j.jpsychires.2016.09.018. 27693981

[8] M. Fava , A. J. Rush , J. E. Alpert , et al., “Difference in Treatment Outcome in Outpatients With Anxious Versus Nonanxious Depression: A STAR*D Report,” American Journal of Psychiatry 165, no. 3 (2008): 342–351, 10.1176/appi.ajp.2007.06111868. 18172020

[9] A. J. Flint and S. L. Rifat , “Two‐Year Outcome of Elderly Patients With Anxious Depression,” Psychiatry Research 66, no. 1 (1997): 23–31, 10.1016/S0165-1781(96)02964-2. 9061801

[10] H. J. Seo , Y. E. Jung , T. S. Kim , et al., “Distinctive Clinical Characteristics and Suicidal Tendencies of Patients With Anxious Depression,” Journal of Nervous and Mental Disease 199, no. 1 (2011): 42–48, 10.1097/NMD.0b013e3182043b60. 21206246

[11] H. Sugawara , T. Tsutsumi , K. Inada , et al., “Association Between Anxious Distress in a Major Depressive Episode and Bipolarity,” Neuropsychiatric Disease and Treatment 15 (2019): 267–270, 10.2147/NDT.S188947. 30697051 PMC6339637

[12] K. Ota , H. Shinzato , N. Otsuka , et al., “Depressive Mixed State and Anxious Distress as Risk Factors for Suicidal Behavior During Major Depressive Episodes,” Scientific Reports 15, no. 1 (2025): 11918, 10.1038/s41598-025-92437-3. 40195461 PMC11976963

[13] K. W. Choi , Y. K. Kim , and H. J. Jeon , “Comorbid Anxiety and Depression: Clinical and Conceptual Consideration and Transdiagnostic Treatment,” Advances in Experimental Medicine and Biology 1191 (2020): 219–235, 10.1007/978-981-32-9705-0_14. 32002932

[14] M. Fava , M. A. Rankin , E. C. Wright , et al., “Anxiety Disorders in Major Depression,” Comprehensive Psychiatry 41, no. 2 (2000): 97–102, 10.1016/S0010-440X(00)90140-8. 10741886

[15] K. Demyttenaere and E. Heirman , “The Blurred Line Between Anxiety and Depression: Hesitations on Comorbidity, Thresholds and Hierarchy,” International Review of Psychiatry 32, no. 5–6 (2020): 455–465, 10.1080/09540261.2020.1764509. 32436448

[16] T. Otsubo , C. Hokama , N. Sano , Y. Watanabe , T. Kikuchi , and K. Tanaka , “How Significant Is the Assessment of the DSM‐5 Anxious Distress Specifier in Patients With Major Depressive Disorder Without Comorbid Anxiety Disorders in the Continuation/Maintenance Phase?,” International Journal of Psychiatry in Clinical Practice 25, no. 4 (2021): 385–392, 10.1080/13651501.2021.1907415. 33840340

[17] M. H. Trivedi , A. J. Rush , S. R. Wisniewski , et al., “Evaluation of Outcomes With Citalopram for Depression Using Measurement‐Based Care in STAR*D: Implications for Clinical Practice,” American Journal of Psychiatry 163, no. 1 (2006): 28–40, 10.1176/appi.ajp.163.1.28. 16390886

[18] D. W. Morris , M. Toups , and M. H. Trivedi , “Measurement‐Based Care in the Treatment of Clinical Depression,” Focus (Am Psychiatr Publ) 10, no. 4 (2012): 428–433, 10.1176/appi.focus.10.4.428.

[19] T. Guo , Y. T. Xiang , L. Xiao , et al., “Measurement‐Based Care Versus Standard Care for Major Depression: A Randomized Controlled Trial With Blind Raters,” American Journal of Psychiatry 172, no. 10 (2015): 1004–1013, 10.1176/appi.ajp.2015.14050652. 26315978

[20] R. H. Hong , J. K. Murphy , E. E. Michalak , et al., “Implementing Measurement‐Based Care for Depression: Practical Solutions for Psychiatrists and Primary Care Physicians,” Neuropsychiatric Disease and Treatment 17 (2021): 79–90, 10.2147/NDT.S283731. 33469295 PMC7813452

[21] M. Zimmerman and J. B. McGlinchey , “Depressed Patients' Acceptability of the Use of Self‐Administered Scales to Measure Outcome in Clinical Practice,” Annals of Clinical Psychiatry 20, no. 3 (2008): 125–129, 10.1080/10401230802177680. 18633738

[22] M. Zimmerman , I. Chelminski , J. B. McGlinchey , and M. A. Posternak , “A Clinically Useful Depression Outcome Scale,” Comprehensive Psychiatry 49, no. 2 (2008): 131–140, 10.1016/j.comppsych.2007.10.006. 18243884

[23] M. Zimmerman , I. Chelminski , D. Young , K. Dalrymple , E. Walsh , and L. Rosenstein , “A Clinically Useful Self‐Report Measure of the DSM‐5 Anxious Distress Specifier for Major Depressive Disorder,” Journal of Clinical Psychiatry 75, no. 6 (2014): 601–607, 10.4088/JCP.13m08961. 24813209

[24] M. Zimmerman , H. Clark , P. McGonigal , L. Harris , C. G. Holst , and J. Martin , “Reliability and Validity of the DSM‐5 Anxious Distress Specifier Interview,” Comprehensive Psychiatry 76 (2017): 11–17, 10.1016/j.comppsych.2017.02.010. 28384524

[25] Y. Aoki , Y. Takaesu , Y. Matsumoto , et al., “A Psychometric Analysis of the Japanese Version of the Clinically Useful Depression Outcome Scale Supplemented With Questions for the DSM‐5 Anxious Distress Specifier (CUDOS‐A),” Neuropsychopharmacology Reports 44, no. 3 (2024): 526–533, 10.1002/npr2.12432. 38838706 PMC11544442

[26] A. J. Rush , M. H. Trivedi , H. M. Ibrahim , et al., “The 16‐Item Quick Inventory of Depressive Symptomatology (QIDS), Clinician Rating (QIDS‐C), and Self‐Report (QIDS‐SR): A Psychometric Evaluation in Patients With Chronic Major Depression,” Biological Psychiatry 54, no. 5 (2003): 573–583, 10.1016/S0006-3223(02)01866-8. 12946886

[27] D. Fujisawa , A. Nakagawa , M. Tajima , et al., “Cross‐Cultural Adaptation of the Quick Inventory of Depressive Symptomatology‐Self Report (QIDS‐SR‐J),” Japanese Journal of Stress Science 25, no. 1 (2010): 43–52.

[28] M. Zimmerman , M. A. Posternak , and I. Chelminski , “Using a Self‐Report Depression Scale to Identify Remission in Depressed Outpatients,” American Journal of Psychiatry 161, no. 10 (2004): 1911–1913, 10.1176/ajp.161.10.1911. 15465991

[29] M. Fava , J. E. Alpert , C. N. Carmin , et al., “Clinical Correlates and Symptom Patterns of Anxious Depression Among Patients With Major Depressive Disorder in STAR*D,” Psychological Medicine 34, no. 7 (2004): 1299–1308, 10.1017/S0033291704002612. 15697056

[30] D. F. Ionescu , M. J. Niciu , E. M. Richards , and C. A. Zarate, Jr. , “Pharmacologic Treatment of Dimensional Anxious Depression: A Review,” Primary Care Companion for CNS Disorders 16, no. 3 (2014): 13r01621, 10.4088/PCC.13r01621. PMC4195641 25317369

[31] N. Sugawara , N. Yasui‐Furukori , T. Tsuji , et al., “The Relationship Between Baseline Clinical Symptom Characteristics and Working Ability in Japanese Patients Treated for Major Depressive Disorder and Painful Physical Symptoms,” Neuropsychiatric Disease and Treatment 16 (2020): 3063–3070, 10.2147/NDT.S274608. 33335397 PMC7737943

[32] R. Inano , N. Sugawara , Y. Kawamata , and N. Yasui‐Furukori , “Predictors of Changes in Depressive Symptoms With Major Depressive Disorder ‐ a Naturalistic 6‐Month Follow‐Up Study,” Neuropsychiatric Disease and Treatment 21 (2025): 1435–1446, 10.2147/NDT.S525179. 40703797 PMC12285889

[33] A. Sato , N. Sugawara , Y. Kawamata , and N. Yasui‐Furukori , “Changes in Suicidal Ideation During Treatment Among Patients With Major Depressive Disorder: A 6‐Month Naturalistic Follow‐Up Study,” Neuropsychopharmacology Reports 44, no. 2 (2024): 371–380, 10.1002/npr2.12428. 38443150 PMC11144608

[34] Y. Ishihara , N. Sugawara , Y. Kawamata , and N. Yasui‐Furukori , “Initial Psychotropic Prescriptions and Symptom Associations in First‐Visit Patients With Major Depressive Disorder: A Single‐Center Cross‐Sectional Study,” Neuropsychopharmacology Reports 45, no. 1 (2025): e12507, 10.1002/npr2.12507. 39568098 PMC11666338

