# Supplementary material for: Symptom‐Domain Correlates of Self‐Reported Anxious Distress in Outpatients With Major Depressive Disorder
Source: Neuropsychopharmacol Rep. 2026 Sep 1;46(3):e70165. doi: 10.1002/npr2.70165 (PMC13534557; doi:10.1002/npr2.70165)
Supplement: Supplementary file 1 [file NPR2-46-e70165-s001.docx]

**Supplementary Material**

Symptom-domain correlates of self-reported anxious distress in outpatients with major depressive disorder

Supplementary Table S1. Correlations between QIDS-J symptom domains and anxious-distress burden (>=3 threshold)

| **QIDS-J domain** | **N** | **Pearson r** | **95% CI** | **P value** | **Spearman rho** | **FDR-adjusted P** |
| --- | --- | --- | --- | --- | --- | --- |
| Sleep | 206 | 0.328 | 0.200 to 0.445 | <0.001 | 0.346 | <0.001 |
| Sad mood | 206 | 0.591 | 0.495 to 0.674 | <0.001 | 0.589 | <0.001 |
| Appetite/weight | 206 | 0.252 | 0.119 to 0.375 | <0.001 | 0.254 | <0.001 |
| Concentration/decision making | 206 | 0.468 | 0.354 to 0.569 | <0.001 | 0.440 | <0.001 |
| Self-view | 206 | 0.517 | 0.409 to 0.610 | <0.001 | 0.532 | <0.001 |
| Suicidal ideation | 206 | 0.508 | 0.399 to 0.603 | <0.001 | 0.498 | <0.001 |
| General interest | 206 | 0.337 | 0.210 to 0.453 | <0.001 | 0.333 | <0.001 |
| Energy/fatigue | 206 | 0.302 | 0.172 to 0.421 | <0.001 | 0.313 | <0.001 |
| Psychomotor | 206 | 0.504 | 0.394 to 0.599 | <0.001 | 0.474 | <0.001 |

Pearson correlations between each of the nine QIDS-J symptom domains and the number of anxious-distress items scored >=3 in the primary analytic cohort (n=206). All correlations were positive and statistically significant after Benjamini-Hochberg false-discovery-rate correction. Values are Pearson r with corresponding P values.

Supplementary Table S2. Correlations between QIDS-J symptom domains and anxious-distress burden (>=2 threshold)

| **QIDS-J domain** | **N** | **Pearson r** | **95% CI** | **P value** | **Spearman rho** | **FDR-adjusted P** |
| --- | --- | --- | --- | --- | --- | --- |
| Sleep | 206 | 0.267 | 0.136 to 0.390 | <0.001 | 0.269 | <0.001 |
| Sad mood | 206 | 0.551 | 0.448 to 0.640 | <0.001 | 0.556 | <0.001 |
| Appetite/weight | 206 | 0.270 | 0.138 to 0.392 | <0.001 | 0.301 | <0.001 |
| Concentration/decision making | 206 | 0.397 | 0.275 to 0.506 | <0.001 | 0.436 | <0.001 |
| Self-view | 206 | 0.459 | 0.343 to 0.560 | <0.001 | 0.446 | <0.001 |
| Suicidal ideation | 206 | 0.441 | 0.324 to 0.545 | <0.001 | 0.445 | <0.001 |
| General interest | 206 | 0.329 | 0.201 to 0.446 | <0.001 | 0.351 | <0.001 |
| Energy/fatigue | 206 | 0.337 | 0.210 to 0.453 | <0.001 | 0.369 | <0.001 |
| Psychomotor | 206 | 0.439 | 0.321 to 0.543 | <0.001 | 0.463 | <0.001 |

Pearson correlations between each QIDS-J symptom domain and the number of anxious-distress items scored >=2 in the primary analytic cohort (n=206). False-discovery-rate correction was applied to account for multiple comparisons. Values are Pearson r with P values.

Supplementary Table S3. Correlations between QIDS-J symptom domains and total anxious-distress score

| **QIDS-J domain** | **N** | **Pearson r** | **95% CI** | **P value** | **Spearman rho** | **FDR-adjusted P** |
| --- | --- | --- | --- | --- | --- | --- |
| Sleep | 206 | 0.319 | 0.191 to 0.437 | <0.001 | 0.326 | <0.001 |
| Sad mood | 206 | 0.622 | 0.531 to 0.699 | <0.001 | 0.607 | <0.001 |
| Appetite/weight | 206 | 0.282 | 0.151 to 0.403 | <0.001 | 0.292 | <0.001 |
| Concentration/decision making | 206 | 0.455 | 0.340 to 0.557 | <0.001 | 0.462 | <0.001 |
| Self-view | 206 | 0.512 | 0.404 to 0.606 | <0.001 | 0.514 | <0.001 |
| Suicidal ideation | 206 | 0.521 | 0.414 to 0.614 | <0.001 | 0.506 | <0.001 |
| General interest | 206 | 0.365 | 0.241 to 0.478 | <0.001 | 0.371 | <0.001 |
| Energy/fatigue | 206 | 0.360 | 0.235 to 0.474 | <0.001 | 0.368 | <0.001 |
| Psychomotor | 206 | 0.527 | 0.421 to 0.619 | <0.001 | 0.512 | <0.001 |

Pearson correlations between each QIDS-J symptom domain and the summed anxious-distress score (range 0-20) in the primary analytic cohort (n=206). All tests were two-sided, and false-discovery-rate correction was applied. Values are Pearson r with P values.

Supplementary Table S4. Distribution of anxious-distress burden across QIDS-J severity categories

| **QIDS-J severity** | **n** | **QIDS-J total, mean ± SD** | **Anxious-distress sum, mean ± SD** | **>=2 threshold, n (%)** | **>=3 threshold, n (%)** | **>=2 count, mean ± SD** | **>=3 count, mean ± SD** |
| --- | --- | --- | --- | --- | --- | --- | --- |
| None/minimal (0-5) | 81 | 2.5 ± 1.7 | 1.8 ± 2.5 | 8/81 (9.9%) | 1/81 (1.2%) | 0.4 ± 0.9 | 0.0 ± 0.3 |
| Mild (6-10) | 71 | 8.0 ± 1.5 | 5.1 ± 4.0 | 32/71 (45.1%) | 6/71 (8.5%) | 1.6 ± 1.7 | 0.4 ± 0.9 |
| Moderate (11-15) | 61 | 12.8 ± 1.4 | 9.5 ± 4.2 | 51/61 (83.6%) | 22/61 (36.1%) | 3.3 ± 1.6 | 1.3 ± 1.6 |
| Severe (16-20) | 47 | 17.9 ± 1.4 | 12.5 ± 4.4 | 44/47 (93.6%) | 34/47 (72.3%) | 4.2 ± 1.4 | 2.6 ± 1.7 |
| Very severe (21-27) | 27 | 22.9 ± 1.9 | 15.9 ± 3.7 | 26/27 (96.3%) | 23/27 (85.2%) | 4.7 ± 0.8 | 3.7 ± 1.4 |

Distribution of anxious-distress burden according to QIDS-J severity categories in the screened cohort. QIDS-J severity categories were defined as follows: <6 (none/minimal), 6-10 (mild), 11-15 (moderate), 16-20 (severe), and >=21 (very severe). Values are presented as number (percentage) or mean ± SD. Anxious-distress burden was defined using both >=2 and >=3 item thresholds.

Supplementary Table S5. Post hoc decomposition of the QIDS-J psychomotor domain

| **Analysis** | **Psychomotor retardation** | **P value** | **Psychomotor agitation** | **P value** |
| --- | --- | --- | --- | --- |
| Correlation with count >=2 | r=0.422 | <0.001 | r=0.359 | <0.001 |
| Correlation with count >=3 | r=0.446 | <0.001 | r=0.438 | <0.001 |
| Correlation with summed score | r=0.476 | <0.001 | r=0.453 | <0.001 |
| Adjusted Poisson model for count >=2 | IRR 1.07 (0.96-1.19) | 0.214 | IRR 1.10 (1.04-1.17) | 0.002 |
| Adjusted Poisson model for count >=3 | IRR 1.06 (0.89-1.27) | 0.505 | IRR 1.24 (1.10-1.39) | <0.001 |

The QIDS-J psychomotor domain is the maximum of the psychomotor retardation and psychomotor agitation items. Correlations are Pearson correlations. Adjusted Poisson models entered psychomotor retardation and psychomotor agitation simultaneously with the other eight QIDS-J domains, age per 10 years, and sex. Estimates are IRRs with 95% CIs.

Supplementary Table S6. Model diagnostics for forced-entry Poisson regression models

| **Model** | **n** | **Residual df** | **Pearson chi-square / df** | **Deviance / df** | **Maximum VIF** |
| --- | --- | --- | --- | --- | --- |
| Count >=2 model | 206 | 194 | 0.96 | 1.25 | 1.82 |
| Count >=3 model | 206 | 194 | 1.17 | 1.23 | 1.82 |

Model fit and possible overdispersion were assessed using Pearson chi-square divided by residual degrees of freedom and deviance divided by residual degrees of freedom. Variance inflation factors were calculated for all predictors entered in the primary forced-entry models.
